# The role of cell–cell and cell–matrix junctional complexes in sebaceous gland homeostasis and differentiation

**DOI:** 10.1186/s12964-024-01835-z

**Published:** 2024-09-23

**Authors:** Aylin Yaba, Torsten Thalheim, Marlon R. Schneider

**Affiliations:** 1 Department of Histology and Embryology, Faculty of Medicine, Yeditepe University, Istanbul, Türkiye; 2 Present Address: Deutsches Biomasseforschungszentrum gGmbH, Torgauer Str. 116, 04347 Leipzig, Germany; 3 Interdisciplinary Centre for Bioinformatics, Härtelstr. 16-18, 04107 Leipzig, Germany; 4 Institute of Veterinary Physiology, University of Leipzig, An den Tierkliniken 7, 04103 Leipzig, Germany

**Keywords:** Sebaceous glands, Sebum, Junctional complexes, Cell–cell adhesion, Cell–matrix adhesion

## Abstract

Sebaceous glands (SG) are essential for maintaining skin integrity, as their lipid-rich secretion (sebum) lubricates and protects the epidermis and hairs. In addition, these glands have an emerging role in immunomodulation and may affect whole-body energy metabolism, besides being an appealing model for research in topics as lipogenesis, stem cell biology and tumorigenesis. In spite of the increasing interest in studying SGs pathophysiology, sebocyte cell–cell and cell–matrix adhesion processes have been only superficially examined, and never in a systematic way. This is regrettable considering the key role of cellular adhesion in general, the specific expression pattern of indivdual junctional complexes, and the reports of structural changes in SGs after altered expression of adhesion-relevant proteins. Here, we review the available information on structural and functional aspects of cell–cell and cell–matrix junctions in sebocytes, and how these processes change under pathological conditions. This information will contribute for better understanding sebocyte differentiation and sebum secretion, and may provide hints for novel therapeutic strategies for skin diseases.

## Background

**Fig. 1:**
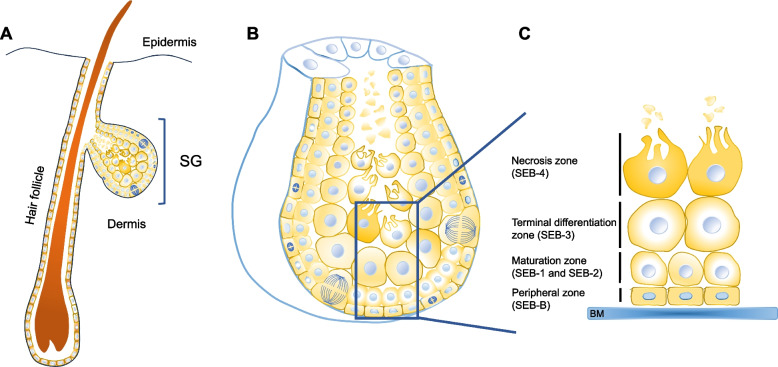
Structure of of the pilosebaceous unit and cellular architecture of the sebaceous gland. **A** Schematic drawing of a pilosebaceous unit in sagittal section. **B** Higher magnification image of the sebaceous gland. The rectangle is enlarged in **C** to demonstrate the peripheral (SEB-B), differentiation (SEB-1–3) and necrosis (SEB-4) zones

The mammalian skin fulfills numerous essential functions by forming a physical, chemical, and biological protective boundary with the external environment, besides acting as a sensory and endocrine organ. It is composed of the epidermis, a constantly renewed stratified epithelium, and the dermis, which harbors skin appendages such as hair follicles, sebaceous glands (SG), and sweat glands within a fibroblast-rich stroma (Fig. 1A). SGs are hair follicle-associated exocrine glands, whose lipid-rich secretion (sebum) primarily lubricates and protects the skin and hairs [1, 2]. In addition to the hair follicle-associated SGs, specialized SGs at specific body sites exert important functions. A prime example is found in the eyelids, where Meibomian glands secret meibum, the lipid fraction of the tear film that is essential for corneal health [3]. Sebum lipids show great variability across different mammals [4], very likely reflecting functional differences. In fury animals, sebum composition adapts to specific needs such as waterproofing, while in humans the sebum’s functions remain controversial. Human sebum has an unusual lipid composition, containing triglycerides, diglycerides, and free fatty acids (57%), wax esters (26%), squalene (12%), and cholesterol (2%) [1]. Wax esters and squalene are typical for sebum and are normally not found elsewhere in the body, and sebum fatty acids show uncommon saturation and branching patterns [5]. Deregulated sebum secretion is a key pathogenic factor in acne, the most common skin disease worldwide and a major burden during adolescence [6, 7] but it is also involved in several other debilitating skin diseases such as atopic dermatitis or psoriasis [8].

However, instead of being simple fat-secreting glands, SGs are adaptable structures that respond to local and systemic stimuli, and constitute an appealing paradigm for research in topics beyond skin biology including stem cell function, adhesion and metabolism [9], the regulation of organ size [10], lipid metabolism [11], host-microbe interaction [12], whole body lipid and energetic metabolism [13] and tumorigenesis [14]. SG cells are interconnected at their lateral and basal surfaces by a variety of junctions that undergo dynamic rearrangements as cells differentiate. In spite of the increasing interest in studying SG pathophysiology, sebocyte cell adhesion processes and the underlying junctional complexes have been only superficially examined, and mostly as a by-product of studies focussing in other skin structures. Thus, systematic studies focussing on the SG are not available despite the general importance of cell–cell and cell–matrix adhesion in biological processes as embryonic development [15], and in particular in the epidermis [16]. In the latter tissue, for instance, corneodesmosomes formed by desmosomal components crosslinked to the cornified envelope bind corneocytes together and are essential for barrier function [16–18]. Importantly, changes in the expression of corneodesmosin, a major component of corneodesmosomes, have been reported in several skin diseases including ichtyoses, psoriasis, atopic dermatitis and benign and maligna skin tumors [19–21]. In addition, severe structural changes in SGs were reported after loss of adhesion-relevant proteins in genetically modified mice [22] (see also Table 1), strongly indicating an essential role for such components in the SG. This essay will highlight studies addressing structural and functional aspects of cell–cell and cell–matrix adhesion in SGs.

**Table 1:** Changes in sebaceous gland morphology and function in genetically modified mice with altered expression of junctional proteins

Junctional protein (Gene symbol)	Type of genetic manipulation	Sebaceous gland changes	References
Corneodesmosin (*Cdsn*)	Deletion in adult skin (KRT14 promoter)	Enlarged glands	[23]
Catenin beta-1 (*Ctnnb1*)	Embryonic, skin-specific expression of a truncated mutant (DN87bcat; KRT14 promoter)	De novo gland formation	[24]
	Postnatal, skin-specific expression of a truncated mutant (DN87bcat; KRT14 promoter)	Impaired sebocyte differentiation and subsequent gland loss	[25]
Claudin-1 (*Cldn1*)	Skin of newborn Cldn1-deficient mice transplanted into nude mice and inducible, skin-specific Cldn1 deletion (KRT14 promoter)	Incomplete sebocyte differentiation and their accumulation in sebaceous ducts	[26]
Protein kinase C iota type (*Prkci*)	Embryonic, skin-specific deletion (KRT14 promoter)	Altered sebocyte differentiation, enlarged glands	[27]
	Embryonic, skin-specific deletion (KRT5 promoter)	Altered sebocyte differentiation, enlarged glands	[28]
Partitioning defective 3 homolog (*Pard3b*)	Embryonic, skin-specific deletion (KRT14 promoter)	Enlarged glands	[29]
Gap junction beta-6 protein (*Gjb6*)	Knockin of the A88V (Clouston syndrome) mutation into the *Gjb6* locus	Enlarged glands	[30]
Gap junction beta-2 protein (*Gjb2*)	Inducible, skin-specific expression of the G45E (KID syndrome) mutation (KRT14 promoter)	Gland atrophy	[31]
Focal adhesion kinase 1 (*Ptk2*)	Embryonic, skin-specific deletion (KRT5 promoter)	Gland hypoplasia	[32]
Tyrosine-protein kinase Fyn (*Fyn*)	Full knockout of *Fyn* and heterozygosity for *Ptk2*	Increased number and size of glands	[33]
Alpha-parvin (*Parva*)	Embryonic, skin-specific deletion (KRT5 promoter)	Enlarged glands	[34]
Embigin (*Emb*)	Full *Emb* knockout	Enlarged glands	[9]
Exostosin glycosyltransferase 1 (*Ext1*)	Inducible, skin-specific *Ext1* deletion (KRT14 promoter) leading to heparan sulfate depletion	Enlarged and irregular glands	[35]

### Sebaceous gland development, morphology and function

SGs are usually found in the skin attached to hair follicles (the pilosebaceous unit), and their secretion coats and moistures the skin and hairs. SG development is intimately connected to hair follicle morphogenesis [36]. Markers of future stem cell compartments, including SRY-box transcription factor 9 (SOX9) and leucine-rich repeats and immunoglobulin-like domain protein 1 (LRIG1), are already expressed early stages of hair follicle formation [37, 38]. As hair follicle morphogenesis and lineage specification progresses, SOX9^+^ keratinocytes migrate towards the future bulge, while LRIG1^+^ cells locate at the upper part of the developing follicle, the future junctional zone [37]. The first sebocytes are generated in the junctional zone by asymmetric cell fate decision of proliferative Lrig1^+^ stem cells [39, 40]. Mature sebocytes are continuously replaced by dividing progenitor cells at the gland’s periphery, but the molecular mechanisms underlying SG cellular turnover remain to be elucidated. While LRIG1^+^ keratinocytes of the junctional zone of the hair follicle are the best-characterized stem cell population linked to SG renewal, sebocytes can also be derived from additional skin stem cell pools, including leucine-rich repeat-containing G-protein-coupled receptor 6^+^ progenitors and keratin 15^+^ stem cells of the upper bulge/isthmus region [37, 38].

Adult SGs (Fig. 1A) usually consist of a single lobule (acinus) or multiple lobules that open into the so-called sebaceous duct, which in turn disembogues into the pilary canal (free sebaceous glands open directly onto the skin’s surface). The glands are enclosed by a connective tissue capsule, formed by a basal lamina and its surrounding extracellular matrix (ECM), that gives rise to a system of trabeculae that separates the various acini and provide structural support [2, 41]. The surrounding stroma is composed of collagenous fibers and fibroblasts, and includes blood vessels and nerves.

Acinar cells show a progressive differentiation (Fig. 1B), which begins at the most peripheral layer and ends in its middle, when lipid-filled sebocytes disrupt and release their cellular contents in a process termed holocrine secretion [2, 42]. Although it is a continuous process, holocrine secretion has been traditionally divided in discrete stages based on the cellular morphology, most commonly in peripheral, maturation, and necrosis zones [41, 43]. More recent studies based on transcriptome data [44, 45] allowed establishing stages with higher resolution (Fig. 1C), including SEB-B for the flat, peripheral and in part mitotically active cells in contact with the basal lamina, SEB-1 and SEB-2 for initial and advanced stage of cells lost contact to the basal lamina and show massive increase in cell volume due to cytoplasmic lipid droplet accumulation, and SEB-3, the most advanced maturation stage, which embraces cells undergoing disruption. A fifth stage (SEB-4) comprising even more advanced cells, but whose transcripts are not assessable anymore, is the immediate source of lipids and cellular debris that reaches the skin surface. This process is guided by fine-tuned changes in the expression of selected genes [2]. From the peripheral layer to the gland’s middle, the transcriptional differentiation programme is initially characterized by proliferation and oxidative phosphorylation, followed by lipid metabolism and finally apoptosis and lysis [45]. One of the best characterized regulators of lipid synthesis in adult sebocytes is stearoyl-CoA desaturase 1 (Scd1), a rate-limiting enzyme in the synthesis of monounsaturated fatty acids, and naturally ocurring or experimentally induced mutations of the *Scd1* gene result in absent or strongly hyoplastic SG and meibomian glands [46].

### General aspects of cell adhesion structures

#### Cell–cell adhesion

**Fig. 2:**
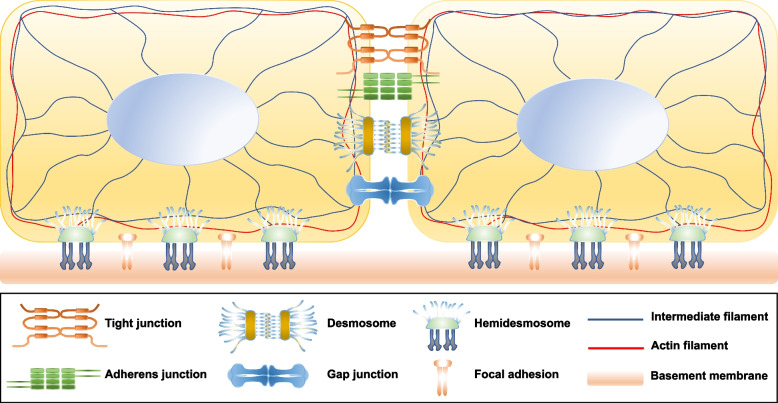
Overview of cell–cell and cell–matrix adhesion structures

Cell–cell adhesion refers to the process of cells sticking to another cell. Adhesion between adjacent cells is mediated by a variety of structures, including tight junctions (TJ), adherens junctions (AJ), desmosomes, and gap junctions (GJ) (Fig. 2).

#### Desmosomes

Desmosomes are intercellular junctions that anchor intermediate filaments to the plasma membrane [47, 48]. They are found primarily in tissues that are subjected to high mechanical stress, such as the myocardium and epidermis, but are also present in the gastrointestinal tract mucosa, the bladder, the meninges, and the dendritic reticulum of lymphatic follicles. The intercellular junctions are composed by proteins from the cadherin family, desmogleins and desmocollins, while on the cytoplasmic side, attachment plaques, including desmoplakins and plakoglobins, secure the intermediate filaments. In addition to its function in cell–cell adhesion, desmosomes perform a variety of roles in processes including cell signaling, differentiation, and tumor formation [49].

#### Adherens junctions

AJ-based extracellular adhesive contacts between cells serve to maintain tissue cohesion, sense and respond to tensile forces at the contact interface, establish cell polarity, and form intracellular links to cytoskeletal elements [50–52]. AJs are capable of localizing proteins to subcellular compartments, which allows for the modulation of signaling pathways. AJs comprise three main components: transmembrane cadherins, armadillo family members, and cytoskeletal adaptor proteins. This core cadherin-catenin complex binds to the actomyosin cytoskeleton and signaling proteins, thereby influencing the overall mechanobiology of cells. In particular, it plays a key role in the integrations of cell proliferation, fate, and positioning to establish the boundary between basal and suprabasal layers of epithelial tissues [53].

#### Tight junctions

TJs, also designated zonulae occludens, seal the paracellular space, thereby creating a permeability barrier that is nearly leak-proof [54]. TJs have two main functions: the fence and gate functions [55]. In terms of the fence function, TJ serves to establish membrane polarity by separating the plasma membrane into apical and basolateral domains, thereby creating an asymmetry regarding the composition of cytosol and plasma membrane proteins and lipids. With regard to the gate function, TJ establish a paracellular diffusion barrier between sealed cells and regulates the passage of solutes and ion selectivity. Furthermore, TJ regulates the organization of cytoskeletal proteins, controlling the actomyosin contractility and distributing cytoskeletal-generated tensional forces [50]. TJ comprises three main transmembrane proteins: junctional adhesion molecules (JAM), claudin, and occludin. Scaffolding proteins, such as zonula occludens proteins (ZO-1, ZO-2, ZO-3), and cingulin associate occludin, claudin, and JAM in tight junctional strands, promoting polymerization.

#### Gap junctions

GJs are essential membrane proteins that regulate the cellular response of heterogeneous cells. They function as intercellular channels of communication, facilitating the transport of small molecules such as amino acids, sugars, intracellular messengers and ions between cells [56]. The fundamental structural units of GJ are connexins. Six connexin proteins are arranged around a pore in a hexagonal configuration, forming a structure called a connexon [57]. Individual connexons (hemichannels) containing a single connexin type are referred to as homomeric, while those comprising different connexins are classified as heteromeric.

#### Cell–matrix adhesion

Cell–matrix adhesion refers to the connection of cells with the extracellular matrix (ECM). This interaction regulates numerous processes beyond cell adhesion, including migration, signaling during morphogenesis, tissue homeostasis, wound healing, and tumorigenesis [58]. Though extremely heterogeneous in its composition, animal ECM largely falls into two categories: the basement membrane (BM) and in interstitial matrix. The BM, a dense thin layer of ECM that marks the boundary of many tissues, consists of laminin and collagen IV networks and crosslinking molecules such as nidogen and perlecan, besides other proteins as fibronectin, tenascin C, fibrillin, agrin, and collagens XV and XVIII. Besides anchoring cellular sheets, the BM has important roles in guiding tissue morphogenesis [59]. In the interstitial matrix, collagens and other proteins such as fibronectin, elastin, laminin, and tenascin, build a characteristic, fibrous network, while glycosaminoglycans, proteoglycans and water contribute to their interstitial spaces [59, 60]. A simplified view of the contribution of these different ECM components to the microenvironmental mechanical properties is that glycosaminoglycans and proteins are responsible for the ECM compressive and tensile strength, respectively [59].

Cell adhesion to ECM substrates is largely mediated by integrin adhesion complexes (IACs), clusters of integrins with associated signaling, scaffolding and cytoskeletal proteins [61]. Integrins are linked via adapter proteins to the actin filaments or intermediate filaments of the own cell, and connect to the BM by binding to a variety of ligands including fibronectin, vitronectin, collagen and laminin. IACs are very heterogeneous and have been classified based on criteria as size, composition, lifetime, cellular distribution and function, and include focal adhesions (FAs), focal complexes, fibrillar adhesions, reticular adhesions, invadosomes, and hemidesmosomes (HDs), among others [61]. The most extensively studied and best characterized cell–matrix adhesions structures are FAs and HDs (Fig. 2). A crosstalk between Fas and HDs integrates mechanotransduction at cell–cell and cell–ECM adhesions and regulates several aspects of cellular behavior, including cell migration and tissue development [62].

#### Cell adhesion in sebaceous glands

**Fig. 3:**
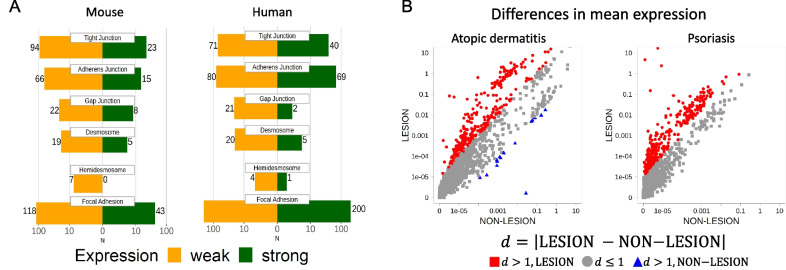
Comparison of gene expression associated to the gene ontology terms on cell–cell interaction {Tight Junction (GO:0070160), Adherens Junction (GO:0005912), Gap Junction (GO:0005921), Desmosome (GO:0030057)} and cell–matrix interaction {Hemidesmosome (GO:0030056), Focal Adhesion (GO:0005925)}. **A** Number of adhesion molecules with weak (yellow) or strong (green) expression (legend: below) in mouse (left) and human (right) sebaceous glands according to single-cell transcription studies. This information is retrieved from existing data published together with our previous study [65]. A gene is strong expressed, if its mean expression over all sebaceous gland samples annotated in the original experimental studies [63, 64] is greater or equal compared to the mean expression of any sample. Gene ontology identifier, terms and corresponding gene sets have been annotated within our previous study [65]. **B** Difference in base-10 logarithmic mean gene expression for matched-pair lesion and non-lesion samples [66]: Atopic dermatitis (left, 4 patients) and psoriasis (right, 3 patients). Each dot represents one gen from one matched-pair, thus redundant gene annotation occurs. Genes with a difference in mean expression greater than 1 between lesion (red squares) and non-lesion (blue triangles) are highlighted (legend: below) for atopic dermatitis and psoriasis. For psoriasis no non-lesion sample with this difference occurs

Numerous cell–cell and cell–matrix adhesion structures have been described in SG cells via a variety of methods including electron microscopy, immunohistochemistry, and immunofluorescence. Recent single-cell transcriptomics studies confirmed the expression of a large number of adhesion-relevant transcripts in both mouse [63] and human [64] SGs. Figure 3A, based on a re-assessment of these data focusing on sebocytes [65], indicates the number of adhesion-relevant transcripts that are expressed at high and low levels in sebocytes compared to other skin cell types. Assessment of another recent dataset based on the spatial transcriptomics analysis of normal and diseased human skin [66, 67] also indicates substantial shifts in the abundance of adhesion-relevant transcripts in psoriasis and atopic dermatitis lesions compared to healthy skin (Fig. 3B).

In the next sections, we will highlight structural and functional roles reported for adhesive structures in SGs.

### Cell–cell adhesion in sebaceous glands

#### Desmosomes

**Fig. 4:**
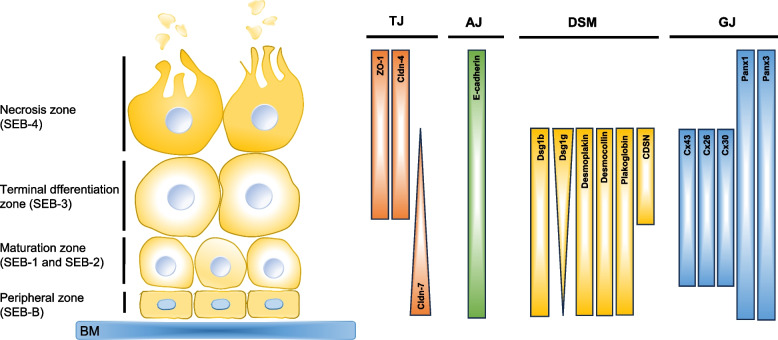
Summary of the localisation of cell–cell adhesion structures in sebocytes at the different zones. SEB: Sebocytes, BM: Basal membrane, TJ: Tight junction, AJ: Adherens junction, DSM: Desmosome, GJ: Gap junction

Various electron microscope-based studies in humans and other species [43, 68–71] reported the presence of desmosomes in SG cells. These reports indicate that desmosomes are quite numerous in basal cells, less frequent in mature cells, and rare or absent in fully differentiated cells (Fig. 4). Several desmosomal components were detected in SGs by immunohistochemistry, including desmogleins, desmoplakin, plakoglobin, and desmocollin. Among these, desmoglein 1 was reported to be found in excretory duct epithelial cells, basal cells, differentiating cells, and mature cells of human SG [72]. Notably, while desmoglein-1-alpha (DSG1A) was completely absent in the SGs of adult mouse ears, DSG1B was expressed in the outer epithelial lining and DSG1G was expressed throughout the gland, including the more differentiated cell layers [73]. Desmoplakin, another major desmosomal protein, stained positive at the cell–cell contacts along the entire SG differentiation axis [26]. Additionally, desmoplakin, desmocollin 3, and placoglobin were found in basal cells, differentiated cells, and mature cells of sebaceous glands [26]. Mutations in genes encoding desmosomal proteins typically result in a spectrum of erosive skin and mucosal phenotypes that may also affect hair or heart [74]. However, changes in the SG have not been commonly reported.

Corneodesmosin (CDSN) is a component of corneodesmosomes, modified desmosomes present in the stratum corneum [18]. Keratin 14-driven Cre-mediated deletion of Cdsn in mice resulted in desmosomal break at the interface between the living and cornified epidermal layers, leading to epidermal tearing upon minor mechanical stress and causing neonatal death. When CDSN was deleted in adult skin, the barrier effect also included hypertrophic sebaceous glands [23]. It remains to be determined whether this represents a compensatory protective mechanism or a direct effect on sebaceous differentiation, as CDSN transcripts are expressed at advanced stages of sebocyte differentiaon and the protein is detectable in terminally differentiated human sebocytes [45] (Fig. 4).

#### Adherens junctions

AJs were not explicitly mentioned in early electron microscopy studies. However, they may have been taken for desmosomes, as both are electron-dense structures surrounded by a fuzzy area [75]. Numerous studies assessed the consequences of E-cadherin loss in the skin in genetically modified mice by employing promoters with a range of cell type-specificity and onset of activity. Collectively, these studies revealed that deletion of E-cadherin caused loss of AJ, altered epidermal differentiation, and severe structural alterations with eventual loss of hair follicles (HF) [22]. Although SG defects have not been explicitly reported, these structures seem to be frequently hypoplastic or absent in the skin of CDH1-deficient mice (see, for instance, [76]). Besides E-cadherin, conspicuous alterations in SGs was reported after skin-specific expression of an N-terminally truncated and stabilized form of the E-cadherin binding partner β-catenin [22]. Expression of the mutant β-catenin during embryonic development resulted in ectopic SGs [24], while postnatal expression caused initial SG duplication followed by inhibition of sebocyte differentiation and loss of SG [25]. These effects, however, are likely related to the role of β-catenin as a key effector of Wnt signaling, rather than to its role in AJs.

#### Tight junctions

Sebaceous glands have long been thought to lack a TJ barrier [43, 77, 78], although they have been described in the central cells of human oral SGs [69]. More recent 2D and 3D observations studies in mouse SGs, however, confirmed the continuous presence of TJP1, also named zonula occludens-1, in the uppermost nucleated sebocytes, thus forming a continuous TJ barrier at a specific single layer of cells with apico-basolateral plasma membrane polarity [26]. This resembles the interfollicular epidermis, where cells in a specific layer of the stratum granulosum are equipped with TJs and show apico-basolateral cell membrane polarity divided at the TJ [54].

Diverse TJ components, including occludin, TJP1, claudins, tricellulin, and angulin-1 were detected in mouse SG in situ and in human SZ95 sebocytes cultured as 3D structures, where they formed a functional TJ barrier [26]. Claudin 4, a subtype of claudins, was found in sebaceous ducts, interfollicular epidermis, and upper two layers of stratified sebocytes [26]. The expression pattern varied, with weak expression observed in sebaceous ducts and the interfollicular epidermis, while strong expression was noted in the upper two layers of stratified sebocytes. Claudin 7, in contrast, did not accumulate in the apical junctions [26] (Fig. 4). Moreover, knockout of claudin 1 caused incomplete degradation of the plasma membrane and nuclei with accumulation of only partly degenerated sebocytes in sebaceous ducts, indicating that the TJ barrier is necessary for holocrine secretion [26]. Another TJ-associated protein is coxsackie- and adenovirus receptor-like membrane protein (CLIMP), also known as adipocyte adhesion molecule (ACAM) [79, 80]. Although its function in sebocytes remains unknown, CLMP is strongly expressed in both human and mouse sebocytes [65].

Cell polarity is regulated by numerous proteins, including the atypical protein kinase (APKC) and the partitioning-defective proteins (PARD) families [81]. Epidermal-specific deletion of aPKCλ caused abnormal hair follicle cycling, progressive losses of hairs and vibrissae, altered differentiation the epidermis, and enlarged sebaceous glands [28]. Loss of Partitioning defective 3 homolog (PARD3B) in mouse skin disturbed the neonatal inside-out skin barrier, probably through compromised polarization and expression of essential tight junction proteins. Notably, PARD3 loss resulted in enlarged SG, most likely a compensatory reaction to rescue the barrier defects seen in newborn mice and ensure a functional barrier during postnatal development via the release of dehydration-counteracting sebum [29].

#### Gap junctions

Already decades ago, GJs were documented mostly between differentiating (mature) sebocytes, but not between peripheral and fully mature cells [43, 77, 78], where they have been suggested to enable the transport of nutrients. GJs are formed by connexins, a family of highly conserved channel-forming proteins that assemble to hexameric hemichannels in the endoplasmic reticulum and Golgi apparatus, before being trafficked to and assembled into the cell membrane [82]. Initial immunohistochemistry studies revealed the presence of only GJA1 (Cx43) [83], but GJB2 (Cx26) and GJB6 (Cx30) are also known to be expressed in SGs [84]. Unique staining patterns were noted for connexin 43 (Cx43), showing faint, dot-like staining in the outer cell layers undergoing no differentiation, contrasted with more pronounced staining observed in the inner layers undergoing differentiation. These findings suggest the existence of multiple gap junctions facilitated by Cx43 within fully developed sebocytes [85] (Fig. 4).

Different mutations in *GJB6* (Cx30) can cause Clouston syndrome in humans, a rare autosomal dominant genetic disorder characterized by alopecia, nail dystrophies, and palmoplantar hyperkeratosis, among other pathologies. A mouse model expressing the *Gjb6* mutation A88V displayed greasy fur and enlarged sebaceous glands [30]. Some mutations in human *GJB2* produce a gain of function and cause syndromic deafness associated with skin disorders, such as keratitis-ichthyosis-deafness syndrome (KIDS). Inducible expression of a mutated *GJB2* (Cx26) that forms constitutively active connexin hemichannels resulted in multiple skin alterations, including SG atrophy while in normal physiological conditions Cx26 shows no staining [31, 85] (Fig. 4).

Pannexins, structurally related to connexins, do not forming intercellular communication channels but rather support the passage of ions and small molecules between the cytoplasm and the extracellular space. Panx1 and Panx3 expression were detected in SGs [86] (Fig. 4), but their role in sebum production or SG homeostasis remains unknown.

#### Cell–matrix adhesion in sebaceous glands

Electron microscope studies revealed that SG peripheral cells connect to the basal lamina via numerous hemidesmosomes [9, 43, 71]. Reflecting the key role of integrins in cell–matrix adhesion, keratinocyte-specific deletion of integrin beta-1 severely disrupted HF and interfollicular epidermis (IFE) structure, and no SG were identifiable in mice seven weeks after birth [87].

Focal adhesion kinase 1 (FAK/PTK2), non-receptor tyrosine kinase that plays important roles in regulating cell adhesion, migration and survival; is a multifunctional protein that can be activated by various cues, including cell adhesion to the extracellular matrix and growth factor signaling [88]. Skin-specific deletion of FAK leads to hair cycle irregularities, moderate epidermal thinning, and sebaceous gland hypoplasia [32]. Surprisingly, exactly the opposite phenotype, this is, enlarged and more numerous SGs have been previously reported in mice heterozygous for PTK2 and additionally carrying a homozygous mutation in tyrosine-protein kinase Fyn (FYN), a non-receptor tyrosine-protein kinase that plays a role in many biological processes including regulation of cell growth and survival, and cell adhesion [33]. Loss of alpha-parvin, an adaptor protein that localizes to FAs and facilitates the interaction of integrins with the actin cytoskeleton, resulted in enlarged SGs [34].

Recently, an important function of the ECM for regulating SG basal progenitor cells and SG metabolism was identified. The transmembrane protein embigin was shown to be specifically expressed in the peripheral SG cells, were it directly binds to the N-terminal fibronectin domain without impairing integrin function [9]. Loss of embigin results in enlarged SG due to cell exit from the progenitor compartment and progression towards differentiation. Notably, embigin regulates basolateral targeting of the monocarboxylate transport SLC16A1, thus coupling adhesion and metabolism [9].

Besides cellular structures, soluble components of the ECM are also essential for SG physiology. Keratinocyte-specific ablation of exostosin glycosyltransferase 1, a key enzyme for the chain elongation step of heparan sulfate biosynthesis, caused hyperplasia of sebaceous glands, leading to exacerbated sebum production and its accumulation on the skin surface of mice, besides other dermatological deffects [35]. In contrast, hyaluronic acid, the most abundant glycosaminoglycan in the extracellular matrix, was reported to have inhibitory effects on SGs. Intradermal injection of HA into hamster auricles decreased both SG size and the level of lipid production, and a double-blind, placebo-controlled, split-face study with oily skin participants revealed that the HA-treated side showed a significant decrease in sebum production [89].

## Conclusions

Cell–cell and cell–matrix junctional complexes emerge from this survey as an essential component of SG development and homeostasis. This conclusion is supported by the specific localization of different junctional structures along the sebocyte differentiation axis (Fig. 4), and by the skin alterations observed after loss of individual adhesion-relevant proteins in mice (Table 1). In this regard, the function of numerous adhesion proteins now known to be expressed in SGs, including CDH13 [90] and CLMP [65], remains to be determined. The identification of embigin as a mediatior of mouse peripheral sebocyte to the extracellular matrix and a regulator of a monocarboxylate transporter [9] demonstrates a direct link between adhesion and lipid metabolism. It remains to be determined whether embigin or other adhesive proteins regulate metabolite flux in human SGs as well. This is of particular interest considering the concept of harnessing of sebum excretion for the therapy of obesity and related disorders [13]. Understanding how peripheral sebocyte aquire blood-derived metabolic substrates, for instance via the solute carrier (SLC) group of membrane transport proteins [91], may provide novel strategies for modulating sebaceous lipogenesis.

Furthermore, the expression of junctional proteins is clearly changed in psoriasis and atopic dermatitis (Fig. 3B), as well as in a variety of other dermatological conditions. Finally, soluble components of the ECM such as hyaluronic acid [89] may also represent an atttractive option in the therapy of diseases characterized by excessive sebum production. Future research, also based on novel in vitro models as SG organoids and innovative methods as single-cell [65] and spatial [45] transcriptomics associated with in situ protein detection, will identify so far unknown cell junctional structures that can regulate sebocyte renewal, proliferation, and differentiation.

These new studies will shed more light on how such changes take place and their role in the disease progress, potentially opening new diganostic, preventive, of therapeutic strategies for selected skin diseases.

## Data Availability

The data presented in Fig. 3 was described previously [65]. The mouse single-cell RNA-seq data [63] and the RNA-seq data from the spatial transcriptomics study [66, 67] can be obtained in the Gene Expression Omnibus repository (GEO, https://www.ncbi.nlm.nih.gov/geo) under the accession number GSE67602 and GSE206391. The human single-cell RNA-seq data [64] are available in the European Genome-Phenome Archive (EGA, https://ega-archive.org) under the accession number EGAS00001002927. The processed gene expression data combining both sources [65] is available in the Leipzig Health Atlas (https://www.health-atlas.de) under the LHA ID 8HEHMJK77T-2.
